# Keeping an Eye on Wild Brown Trout (*Salmo trutta*) Populations: Correlation Between Temperature, Environmental Parameters, and Proliferative Kidney Disease

**DOI:** 10.3389/fvets.2019.00281

**Published:** 2019-08-22

**Authors:** Aurélie Rubin, Pauline de Coulon, Christyn Bailey, Helmut Segner, Thomas Wahli, Jean-François Rubin

**Affiliations:** ^1^Department of Infectious Diseases and Pathobiology, Centre for Fish and Wildlife Health, University of Bern, Bern, Switzerland; ^2^La Maison de la Rivière, Tolochenaz, Switzerland; ^3^Land, Nature, Environment Institute, University of Applied Sciences and Arts Western Switzerland, Geneva, Switzerland; ^4^Department of Ecology and Evolution, Faculty of Biology and Medicine, University of Lausanne, Lausanne, Switzerland; ^5^Fish Immunology and Pathology Laboratory, Animal Health Research Center (CISA-INIA), Madrid, Spain

**Keywords:** proliferative kidney disease, *Salmo trutta*, *Tetracapsuloides bryosalmonae*, water temperature, water quality, ecomorphology, wild fish population, aquatic fieldwork

## Abstract

Proliferative kidney disease (PKD) is an emerging disease of salmonids caused by the myxozoan parasite *Tetracapsuloides bryosalmonae*, which plays a major role in the decrease of wild brown trout (*Salmo trutta*) populations in Switzerland. Strong evidence demonstrated that water temperature modulates parasite infection. However, less knowledge exists on how seasonal water temperature fluctuations influence PKD manifestation under field conditions, how further environmental factors such as water quality may modulate the disease, and whether these factors coalesce with temperatures role possibly giving rise to cumulative effects on PKD. The aims of this study were to (1) determine the correlation between seasonal course of water temperature and PKD prevalence and intensity in wild brown trout populations, (2) assess if other factors such as water quality or ecomorphology correlate with the infection, and (3) quantitatively predict the implication of these factors on PKD prevalence with a statistical model. Young-of-the-year brown trout were sampled in 45 sites through the Canton of Vaud (Switzerland). For each site, longitudinal time series of water temperature, water quality (macroinvertebrate community index, presence of wastewater treatment plant effluent) and ecomorphological data were collected and correlated with PKD prevalence and intensity. 251 *T. bryosalmonae*-infected trout of 1,118 were found (overall prevalence 22.5%) at 19 of 45 study sites (42.2%). Relation between PKD infection and seasonal water temperature underlined that the mean water temperature for June and the number of days with mean temperature ≥15°C were the most significantly correlated parameters with parasite prevalence and intensity. The presence of a wastewater treatment plant effluent was significantly correlated with the prevalence and infection intensity. In contrast, macroinvertebrate diversity and river ecomorphology were shown to have little impact on disease parameters. Linear and logistic regressions highlighted quantitatively the prediction of PKD prevalence depending on environmental parameters at a given site and its possible increase due to rising temperatures. The model developed within this study could serve as a useful tool for identifying and predicting disease hot spots. These results support the importance of temperature for PKD in salmonids and provides evidence for a modulating influence of additional environmental stress factors.

## Introduction

Beginning in the 1980s, the catch of brown trout *Salmo trutta* in Switzerland experienced a massive decline of up to 50% (1–4). This process is still ongoing. Hence, the wild salmonid populations are considered to be threatened (5–7). Investigations within a nationwide project suggested the decrease was due to multifactorial drivers (2). Among the parameters involved, a parasitic disease of salmonids, proliferative kidney disease (PKD) was considered to play a major role (2, 3, 8–10).

The causative agent of PKD is the myxozoan parasite *Tetracapsuloides bryosalmonae* (11, 12). The life cycle of the parasite comprises salmonid fish species as vertebrate hosts and bryozoans, mainly *Fredericella sultana* (Blumenbach), as invertebrate hosts (13–16). Spores, which have developed in bryozoans, are released into the water and upon contact with a suitable vertebrate host, infect the fish through skin and gills (14, 17). Via the circulatory system, the parasites eventually reach the target organs, mainly the kidney. The infection may cause renal swelling (18–20). In the kidney, the parasite normally develops sporogonic stages in the tubuli, from where spores are released via the urine and can infect bryozoans again (15, 21, 22). Depending on host susceptibility and seasonal conditions, extrasporogonic stages might instead proliferate in the interstitial tissue causing PKD, which may lead to high mortality rates (18, 19, 23). Juvenile fish getting in contact with the parasite for the first time appear to be particularly susceptible to the infection and to PKD pathogenesis (8, 19, 24, 25).

Several freshwater fish diseases are suggested to be sensitive to the rising temperatures associated with climate change (26–30). Also for PKD, water temperature is a key parameter for the disease prevalence and intensity for Swiss wild brown trout populations (8–10, 31), wild Atlantic salmon *Salmo salar* L. in Norway (32), and brown trout (33) and rainbow trout *Oncorhynchus mykiss* (Walbaum) from laboratory experiments (34–37) as examples. Renal pathology and trout mortalities are enhanced at water temperatures of ≥15°C (19, 23, 25, 38, 39). Consequently, PKD-associated mortalities and disease symptoms show a seasonal occurrence and appear to be most pronounced during summer and early autumn (8, 32, 34, 40). Temperature could act directly or indirectly on this host-parasite system. Indeed, high temperature could promote spore production by bryozoans (41), accelerate parasite proliferation in fish (18, 23, 36), modulate the host immune response (37) and modify the parasite transmission opportunities (33).

In addition, freshwater fish diseases can be influenced by a multitude of stressors besides temperature (27, 42, 43), such as habitat quality, water levels, pH, and water quality (26, 44, 45). This last factor might indeed have an influence on PKD prevalence, intensity and mortality as previously investigated (31, 46, 47).

To date, data on the influence of temperature on *T. bryosalmonae* infection of fish is largely derived from laboratory experiments using constant temperature(s). In the wild, the influence of long-term water temperature measurements on the development of the disease has rarely been investigated. Besides temperature, other environmental factors have been individually revealed to influence *T. bryosalmonae* infection, but seldom in a combined way. In addition, rainbow trout is often used as a model species for laboratory investigations of fish disease, including to reproduce PKD in controlled conditions [e.g., (14, 17, 18, 23, 34–37)]. Though, rainbow trout and brown trout react differently to *T. bryosalmonae* infection, in terms of sensitivity to infectious agent and environmental stress (46), intensity of *T. bryosalmonae* infection (48), or temperature-dependant modulation of the parasite (49) for example. Therefore, some of the conclusions based on experiments with rainbow trout might not correspond to the situation of wild brown trout populations in their natural habitat with fluctuating temperatures. Thus, the influence of the course of water temperature over time, with its seasonal, local and diurnal variations, on PKD infection in wild brown trout populations need to be investigated. Moreover, the possible cumulative effects of other environmental factors and their predicted consequences on the disease should also be taken under consideration. Field investigations are therefore of crucial importance for a better understanding of the brown trout—*T. bryosalmonae* host parasite system and possible future implications by the global warming on population dynamics.

The aims of the present study were to (1) determine the correlation between seasonal course of water temperature and PKD prevalence and intensity in wild brown trout populations, (2) identify if additional environmental parameters, such as water quality or ecomorphology, might have cumulative effects on PKD infection, and (3) quantitatively predict the combined consequences of these environmental parameters on the disease prevalence.

## Materials and Methods

### Study Sites

**Figure 1:**
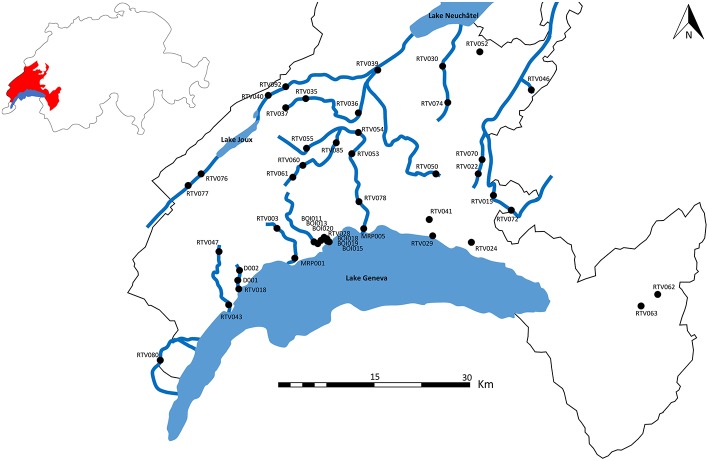
Study sites in the canton of Vaud (Switzerland). Canton of Vaud is shown in red. Rivers comprising at least two sampling sites are shown.

Forty five stations located over 18 rivers of the Canton of Vaud in Switzerland were analyzed (Figure 1). For each station, data on *T. bryosalmonae* infection status of fish (prevalence and intensity), water temperature, biological water quality assessed through macroinvertebrate sensitivity, presence/absence of a wastewater treatment plant (WWTP) and ecomorphology were collected (Table 1).

**Table 1:** Study sites, PKD status, temperature, and environmental data.

	**PKD data**				**Environmental data**			**Temperature data**				
**Site index**	**Number of infected fish**	**PKD prevalence (%)**	**Infection degree**	**Alteration degree**	**IBCH note**	**Upstream WWTP**	**Ecomorphology**	**Mean June [^**°**^C]**	**Mean Summer (June-July-August) [^**°**^C]**	**Days with daily mean ≥ 14^**°**^C**	**Days with daily mean ≥ 15^**°**^C**	**Days with daily mean ≥ 16^**°**^C**
BOI011	0	0	0.0	0.0	13	Yes	1	13.9	14.5	62	35	4
BOI013	0	0	0.0	0.0	9	Yes	1	14.7	15.1	72	54	24
BOI020	0	0	0.0	0.0	9	No	1	15.0	16.2	85	82	56
RTV028	3	13	3.7	3.7	14	Yes	1	15.3	15.6	83	65	38
BOI018	17	68	3.8	3.5	12	Yes	1	15.0	15.8	84	81	41
BOI019	22	88	4.6	4.4	7	Yes	1	15.1	15.9	85	80	47
BOI015	22	88	4.6	4.4	12	Yes	1	15.7	16.2	86	83	60
D002	0	0	0.0	0.0	8	No	4	15.2	16.3	86	83	68
D001	0	0	0.0	0.0	7	No	1	12.6	13.3	11	1	0
RTV018	0	0	0.0	0.0	6	No	1	11.8	11.3	4	0	0
RTV063	0	0	0.0	0.0	13	No	1	8.5	8.6	0	0	0
RTV062	0	0	0.0	0.0	13	No	1	9.3	9.3	0	0	0
RTV003	0	0	0.0	0.0	14	Yes	2	10.2	7.3	0	0	0
MRP001	0	0	0.0	1.7	17	Yes	1	10.6	9.4	0	0	0
RTV072	1	4	3.0	3.0	17	Yes	1	15.3	15.0	70	50	20
RTV015	1	4	3.0	2.0	17	Yes	1	15.3	15.0	68	50	20
RTV022	8	31	4.4	4.4	12	No	1	14.1	14.4	63	31	10
RTV070	6	24	3.7	3.8	12	No	1	14.4	14.6	67	40	12
RTV046	0	0	0.0	0.0	11	No	1	13.3	14.1	55	19	1
RTV074	24	96	3.0	2.6	11	Yes	1	15.0	15.1	70	55	26
RTV030	12	48	1.8	2.0	15	Yes	1	15.9	15.9	79	67	51
RTV037	0	0	0.0	0.0	15	No	1	11.1	9.7	0	0	0
RTV035	0	0	0.0	0.0	13	Yes	1	13.4	12.4	16	5	0
RTV036	0	0	0.0	0.0	13	Yes	3	14.2	13.5	41	14	1
RTV077	10	38	3.3	2.7	17	No	1	17.5	16.3	79	64	49
RTV076	19	70	3.0	3.1	16	No	2	15.3	13.8	38	30	15
RTV040	0	0	0.0	0.0	14	No	1	10.0	9.7	0	0	0
RTV092	0	0	0.0	0.0	15	No	2	NA	NA	NA	NA	NA
RTV039	1	4	3.0	2.0	8	Yes	4	15.0	13.7	45	31	7
RTV055	0	0	0.0	0.0	14	Yes	3	11.8	10.3	0	0	0
RTV054	8	32	2.3	2.3	14	Yes	2	14.4	13.2	33	12	2
RTV053	23	92	3.7	3.7	13	Yes	4	16.5	15.0	62	48	33
RTV078	9	38	2.8	3.2	8	Yes	1	16.3	15.5	84	59	35
MRP005	18	72	3.9	3.9	9	Yes	2	18.0	16.7	90	74	59
RTV061	0	0	0.0	0.0	11	Yes	1	13.6	12.7	14	6	1
RTV060	21	81	4.0	4.2	15	Yes	1	14.5	13.2	39	13	6
RTV085	26	100	3.7	3.8	15	Yes	1	14.0	13.8	47	23	3
RTV024	0	0	0.0	0.0	10	No	1	15.0	15.1	82	50	17
RTV029	0	0	0.0	0.0	8	No	1	15.3	15.2	71	53	28
RTV041	0	0	0.0	0.0	NA	No	2	14.7	14.7	67	44	20
RTV043	0	0	0.0	0.0	16	No	1	13.6	12.5	17	2	0
RTV047	0	0	0.0	0.0	13	No	1	13.2	13.5	33	2	0
RTV050	0	0	0.0	0.0	15	No	1	13.0	13.1	25	3	0
RTV052	0	0	0.0	0.0	10	No	1	12.3	13.3	22	3	0
RTV080	0	0	0.0	0.0	10	No	1	7.8	7.7	0	0	0

*NA, no data for this site*.

### Temperature Data

Water temperature was measured with temperature loggers (HOBO® Water Temp Pro v2 Data Logger, Onset, Cape Cod Massachusetts, USA) recording the water temperature every 15 min. Before placing the loggers into the water and upon sampling, they were compared to a reference thermometer to determine possible drifts which could then be considered for the temperature values. Temperature measurements were selected from the 1st of March 2014, which was considered as the fry emergence date for streams of the Midlands (Rubin, pers. obs.), until the 31st of August 2014 since fish were sampled at the beginning of September. For two sites (RTV063 and RTV022), water temperatures were extrapolated with upstream or downstream loggers. No temperature data were available for RTV092.

Six types of temperature values are described in the study: (1) the monthly mean temperature from March to August, (2) the mean temperature from the “spring” period (March–May), the mean temperature from the “summer” period (June–August) and the total mean during the whole period, (3) the maximum temperature (absolute maximum and daily mean maximum), (4) the number of days with a daily mean temperature ≥ 13°C to ≥ 19°C, and (5) the degree days, calculated as the sum of the daily mean temperature values from the 1st of March to the 31st of August 2014.

### Additional Environmental Factors

#### Macroinvertebrate Analysis

A good indicator for the assessment of water quality and ecological quality is the macroinvertebrate community, depending on the observed taxa and their sensitivity to pollutants (50–56). The standardized method “IBCH” (Swiss biological index) developed by the Swiss Federal Office for the Environment (53) is used to determine the biological water quality and ecological status through assessment of the macroinvertebrate community. This method, also applied in Bailey et al. (31), was performed at all 45 study sites except the station RTV41, where water was loaded with a huge amount of sediment due to construction works preventing sample collection. For an assessment of the biological water quality during the life period of sampled fish (March to September 2014), macrozoobenthos samplings were performed between the 12th of March and 22nd of April 2015, following the favorable sampling periods in function of the altitude, defined by the method (53). 35 macroinvertebrates samplings were performed by our team and eight stations were analyzed by collaborators of the department of water protection from the General Directorate of the Environment from the canton of Vaud.

This standardized method (53) consisted of eight macroinvertebrate samplings with a normalized net in function of the substratum and waterflow. After capture, all material was fixed in a container filled with 85% ethanol. In the laboratory, the material was sorted and determined using a binocular loupe. The determination of each individual was performed until family taxonomic level, using the reference book of Tachet et al. (50). Abundance of all taxa was recorded. Finally, the IBCH index, a score between 0 (very low water quality) and 20 (excellent), was calculated based on the diversity of observed taxa and their sensitivity to water quality. Based on this index, a water quality class was assigned to each station, with the scale: very good water quality = index 17–20, good = 13–16, medium = 9–12, poor = 5–8, bad = 0–4. The macrozoobenthos samplings were performed relating to ethical approvals (permission delivered by the Service of Hunting, Fishing and Surveillance of the Biodiversity and Landscape Division from the General Directorate of the canton of Vaud).

#### Wastewater Treatment Plant

The presence/absence of a wastewater treatment plant effluent upstream of the study site was also determined using topographic maps.

#### Ecomorphology

Ecomorphology is also a potential stressor affecting fish health (57–59) and fish density in streams (60–62). The ecomorphology was determined using the standardized method “Ecomorphology” from the Swiss Federal Office for the Environment (63). This method aims to determine the structural diversity of the stream, its connectivity and interactions with the surrounding area. A class was attributed to each of 45 river sections depending on the state of the riverbed, riverbank and shore, following the scale 1 = natural, 2 = little affected, 3 = very affected, 4 = artificial, 5 = underground pipe. These data were obtained from the geodatabase from the Federal Office of Topography (Swisstopo) (64).

### Fish Analysis

Twenty five young-of-the-year (YOY) brown trout were sampled by electrofishing at each site, resulting in a total number of 1,118 fish. The sampling campaign took place over a period of 10 days, between the 1st and 11th of September 2014. This period was chosen based on previous PKD studies in Swiss streams (8–10) and PKD analysis in the river Boiron de Morges (Rubin, unpublished data), showing that the peak of *T. bryosalmonae* infection in brown trout occurs in late August, beginning of September. After this period, typical PKD kidney alterations could still be visible but parasites might already have been cleared out by the immune system of the fish or have left the fish, causing therefore difficulties for the diagnosis (10).

After electrofishing, juvenile brown trout (total body length 40–118 mm, mean of 76 mm ± standard deviation of 13 mm) were euthanized with an overdose of MS222 (3-aminobenzoic acid ethyl ester, 300 mg l-1, Argent Chemical Laboratories). The fish body cavity was opened, and whole animals were fixed in 4% buffered formalin until later kidney removal in the laboratory. Whole fixed kidneys were embedded in paraffin. Organs were cut using routine histological methods and stained with haematoxylin and eosin (H&E). One section of the full length of the kidney was prepared per fish. Thus, one slide per fish was examined for the presence of *T. bryosalmonae*. The degree of infection (infection intensity corresponding to an estimation of the numbers of observed parasites) and the alteration degree (tissue proliferation due to pathological alterations) were recorded for each sample using a microscope. For each slide a common score system from 0 (no parasite/no alteration) to 6 (at least 10 parasites per high power field with 400x magnification/severe alteration) was applied as in Bettge et al. (18) and Schmidt-Posthaus et al. (39). If at least one parasite was observed, the slide was considered as histologically positive. The infection/alteration degree per station were then calculated as the mean of all infection/alteration scores from a site. The prevalence of PKD (%) was calculated as the percentage of *T. bryosalmonae* infected individuals within all sampled animals at a particular site.

Histology was chosen for PKD diagnosis instead of Polymerase Chain Reaction (PCR) due to time, material and crew resources in the field. This method also permits to assess the severity of pathological response and the state of the disease. However, histological examination may have missed the parasites for fish with very low infection levels. Thus, immunohistochemistry was performed on three randomly selected fish per station where no *T. bryosalmonae* parasites were observed on any H&E stained slide, in order to corroborate negative results. Therefore, immunohistochemistry was performed on 78 samples using an anti-*T. bryosalmonae* (PKX) monoclonal antibody (AquaMab-P01, Aquatic Diagnostic Ltd., Stirling, Scotland) applying the method described by Bettge et al. (18).

Fish samplings were performed relating to ethical approvals (permission number VD2871 delivered by the Service of Consumption and Veterinary business of the canton of Vaud).

### Statistical Analysis

The sample size used for the statistical analysis, contains 1,068 samples. Data of sites RTV041 and RTV092 were excluded because of the absence of, respectively, IBCH index and temperature data.

The software RStudio (version 1.1.463) was used to perform the statistical analysis. An univariate analysis was conducted using Pearson's coefficient to investigate the correlation between the prevalence of *T. bryosalmonae* infected fish, infection degree, alteration degree and temperature data, as well as potentially aggravating environmental parameters. Student's *t*-test was also applied to test the significant differences (*p* < 0.05) in the means of temperature variables and other environmental factors between *T. bryosalmonae* positive fish and PKD healthy trout from all sampling sites.

Multivariate analysis was applied using a linear probability model (LPM) and a logistic model (Logit) that quantified the implication of the different parameters on the PKD infection. The following equation was applied for the linear regression:

$$\begin{array}{l} PKD_{i}=\beta_{0}+\beta_{1}*Temp_{j}+\beta_{2}*IBCH_{j}+\beta_{3}*WWTP_{j}\\ \;+\beta_{4}*Ecomorphology_{j}+\epsilon_{i} \end{array}$$

Where:

*PKDi* is a binary variable taking the value of 1 if the trout *i* is infected with *T. bryosalmonae*, and 0 otherwise,
*Temp* is an indicator of temperature,
*IBCH* is the value of the IBCH index,
*WWTP* is a binary variable with a value of 1 if a WWTP is present upstream the study site, and 0 otherwise,
*Ecomorphology* is a categorial variable (from 1 for a natural section to 4 for a very anthropized sector),
€ is the error term or the residual variation that the model cannot explain,
Index *i* represents variable at the trout level and index *j* represents variable at the site level.

The average marginal effect was applied for the Logit model, in order to get comparable results and not “log odd ratio” data. The conditional expectation was also applied to predict the variation of PKD prevalence due to temperature, while controlling the other environmental factors.

## Results

### Water Temperature

A selection of the temperature results is given in Table 1 (all temperature data are given in Supplementary Material). Highest mean temperature during the study period (March to August 2014), and during the summer (June to August) was observed at the outlet of the river Venoge (MRP005) with a value of, respectively, 13.8 and 16.7°C. Mean temperature of June reached <10°C in three stations, was comprised between 10 and 15°C in 28 cases, and surpassed 15°C in 13 stations. The warmest daily mean temperature (20.6°C) was observed in the Orbe (RTV077) and the Venoge (MRP005). Some stations never reached a daily mean temperature of ≥15°C, while a maximum of 83 days was measured in the Boiron (BOI015) and the Dullive (D002). The degree days varied from 1,090 (RTV063) to 2,539 (MRP005).

### Additional Environmental Factors

IBCH scores were calculated for each site (Table 1) based on the sensitivity and abundance of macroinvertebrate taxa. The lowest obtained IBCH score was 6 (RTV018) and the highest IBCH score obtained was 17 (MRP001, RTV015, RTV072, and RTV077). Four stations corresponded to the water quality class “very good,” 20 belonged to the class “good,” 13 sites were classified as “medium” and seven were in the class “poor.” Thus, all water quality classes were represented in the samples, except the poorest class.

A WWTP was present upstream at 24 of the study sites (53.3%) (Table 1). Among them, 15 stations were assessed as PKD-positive (62.5%), with prevalence ranging from 4 to 100% (mean of 52 ± 34%) of *T. bryosalmonae* infected fish.

The ecomorphology class indicates the condition of the riverbed. Thirty four stations belonged to the class 1, six were in the class 2, two belonged to class 3 and three were considered as anthropized (class 4) (Table 1). No site belonged to the class 5.

### PKD Manifestation

**Figure 2:**
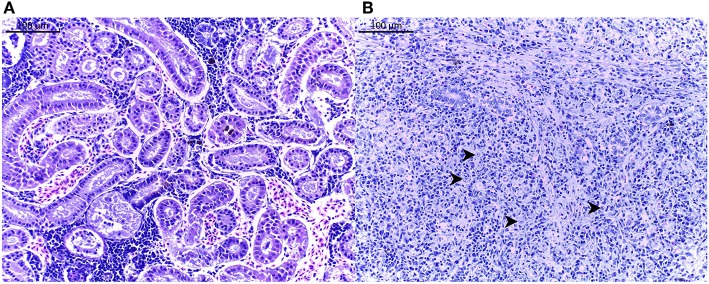
Histological pictures of brown trout *Salmo trutta* posterior kidney. **(A)** Histological assessment of an infection score of 0 (no parasite). **(B)** Histological assessment of an infection score of 6 (severe infection). Some parasites *Tetracapsuloides bryosalmonae* are shown (arrows). Scale bar = 100 μm. Pictures are taken from H&E stained slides.

Stocking of fry or juvenile brown trout for sustaining natural populations was performed since several years in 16 study sites (stocking performed at a maximum of 2 km upstream the study site). Thus, for a comparison of wild fish between all sites, no stocking took place before our fish sampling in 2014, in agreement with the fish inspectorship of the canton of Vaud. All YOY caught here originate therefore from natural spawning. Among the 45 sampled sites, 19 stations (42.2%) were assessed as *T. bryosalmonae*-positive (Table 1). The disease was present in the rivers Boiron de Morges, Broye, Flon de Carrouge, Mentue, Orbe, Venoge, and Veyron. Presence of *T. bryosalmonae* was observed in 251 brown trout from the 1,118 sampled animals (22.5%). The observed prevalence ranged from 0 to 100%. Among the 19 PKD-positive sites, the highest site-specific infection degree (score of 4.64) was found in the Boiron (BOI019), while the lowest (score of 1.84) was seen in the Mentue (RTV030) (Figure 2). However, even if PKD prevalence was low ( ≤ 10%), infected fish showing high infection degree (≥3.0) were found. When considering the degree of alteration, mean values between 1.7 to 4.4 at sites with infected fish were found.

### Correlation of Environmental Variables and PKD

Pearson's correlation coefficient between *T. bryosalmonae* prevalence/infection degree/alteration degree and temperature parameters/other environmental variables is given in Table 2. A positive linear relation appeared between infected fish/infection degree/alteration degree and every temperature variable but remained rather low (*r* < 0.5). The strongest correlation was obtained with the mean temperature of June (*r* = 0.406 with prevalence, 0.366 with infection degree, 0.351 with alteration degree), followed by the number of days with a daily mean temperature ≥15°C (N days ≥ 15) (*r* = 0.373 with prevalence, 0.363 with infection degree, 0.337 with alteration degree). No correlation was found between PKD data and IBCH index (*r* = 0.044 with prevalence, 0.002 with infection degree, −0.001 with alteration degree) and ecomorphology (*r* = 0.041 with prevalence, 0.026 with infection degree, 0.042 with alteration degree), in contrast to the presence of an upstream WWTP (*r* = 0.321 with prevalence, 0.303 with infection degree, 0.295 with alteration degree). Site-specific infection degree and prevalence of infected fish were positively correlated (*r* = 0.808), as well as alteration degree and prevalence of infected fish (*r* = 0.750). Infection degree and alteration degree were strongly correlated (*r* = 0.952).

**Table 2:** Pearson's correlation between *T. bryosalmonae* infected brown trout prevalence, infection degree, alteration degree, and variables.

**Variables**	**PKD prevalence**	**Infection degree**	**Alteration degree**
Mean temperature of march [°C]	0.069	0.091	0.098
Mean temperature of April [°C]	0.209	0.209	0.199
Mean temperature of May [°C]	0.311	0.296	0.286
Mean temperature of June [°C]	0.406	0.366	0.351
Mean temperature of July [°C]	0.304	0.289	0.267
Mean temperature of August [°C]	0.307	0.297	0.276
Mean temperature of spring (March–May) [°C]	0.213	0.216	0.211
Mean temperature of summer (June–August) [°C]	0.346	0.325	0.304
Total mean temperature [°C]	0.306	0.294	0.279
Maximum temperature [°C]	0.146	0.114	0.106
Maximum daily mean temperature [°C]	0.315	0.307	0.301
Days with daily mean temperature ≥ 13°C	0.315	0.298	0.282
Days with daily mean temperature ≥ 14°C	0.358	0.343	0.322
Days with daily mean temperature ≥ 15°C	0.373	0.363	0.337
Days with daily mean temperature ≥ 16°C	0.360	0.350	0.323
Days with daily mean temperature ≥ 17°C	0.308	0.288	0.266
Days with daily mean temperature ≥ 18°C	0.224	0.202	0.187
Days with daily mean temperature ≥ 19°C	0.150	0.138	0.116
Degree days	0.307	0.295	0.277
IBCH note	0.044	0.002	−0.001
WWTP	0.321	0.303	0.295
Ecomorphology	0.041	0.026	0.042

### Average Differences

**Figure 3:**
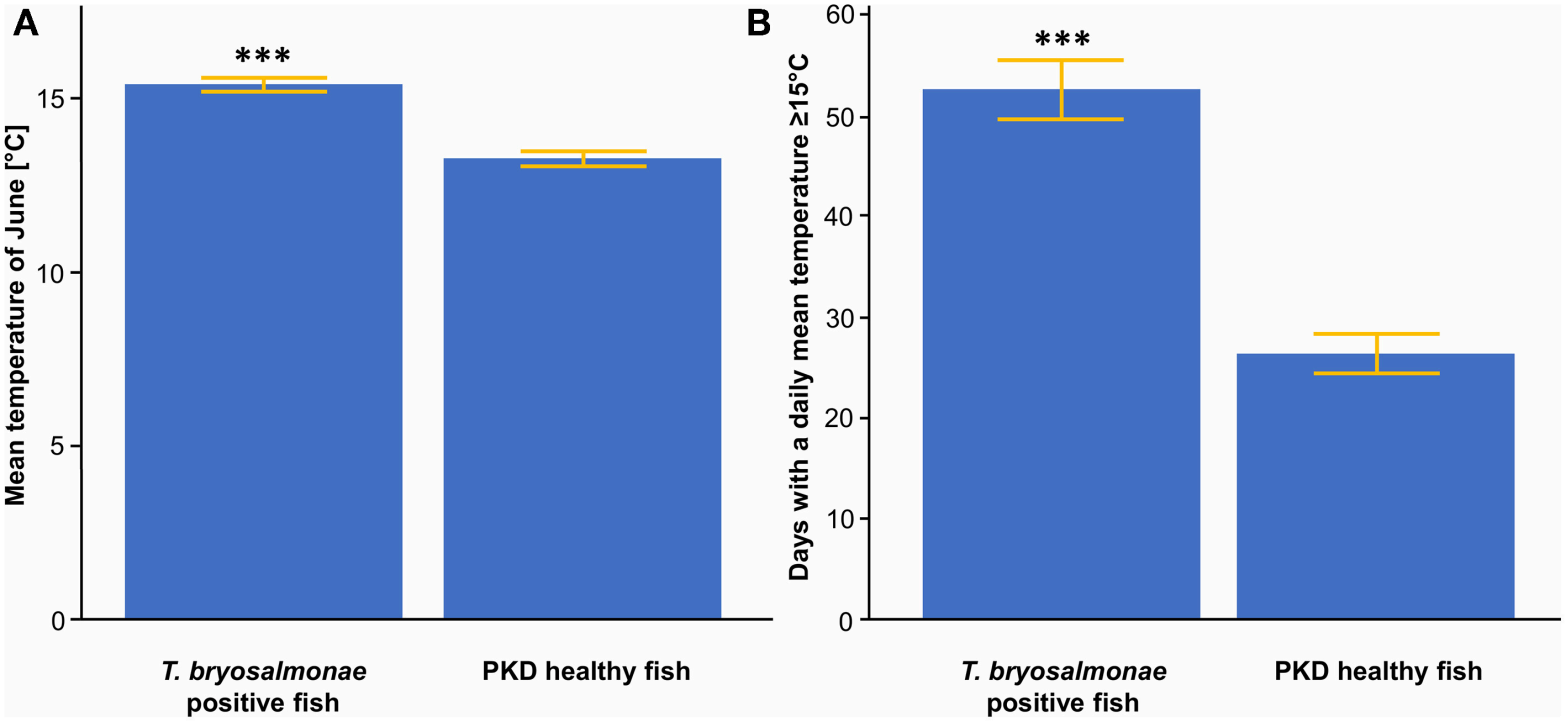
**(A)** Mean temperature of June between *T. bryosalmonae* infected and PKD healthy fish. **(B)** Number of days with a daily mean temperature ≥ 15°C between *T. bryosalmonae* infected and PKD healthy fish. Yellow lines indicate standard error, asterisks indicate levels of significance (*t*-Test), ^***^p < 0.001.

Significant differences in the means of temperature parameters between sites with infected and not infected fish were observed. Mean temperature of June was significantly higher (mean of 15.4°C) for sites with infected fish than for sites with trout without parasites (mean of 13.4°C) (Figure 3A). The same applied for the N days ≥ 15 (Figure 3B).

**Figure 4:**
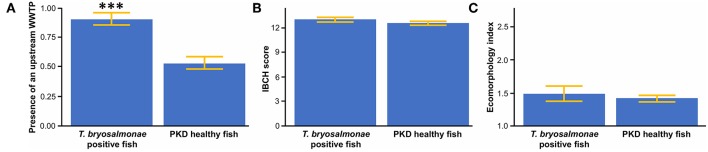
**(A)** Mean presence of an upstream wastewater treatment plant (WWTP) between *T. bryosalmonae* infected and PKD healthy fish. **(B)** Mean biological score (IBCH) between *T. bryosalmonae* infected and PKD healthy fish. **(C)** Mean ecomorphology index between *T. bryosalmonae* infected and PKD healthy fish. Yellow lines indicate standard error, asterisks indicate levels of significance (*t*-test), ^***^p < 0.001.

The presence of an upstream WWTP significantly positively influenced the PKD prevalence (Figure 4A). On the contrary, no significant difference appeared between sites with infected and sites with parasite-free fish for IBCH score (Figure 4B) and ecomorphology (Figure 4C).

### Quantification of the Predicted Effect of Temperature and Additional Environmental Factors on PKD Prevalence

Two models, a linear model (LPM) and a logistic model (Logit), were compared to quantify the predicted variation of PKD prevalence due to an increase of one unit of the temperature variables and other environmental factors. These analyses were performed by separating the temperature variables in two categories (temperature means and N days ≥ x°C), which permit an estimate of the best temperature indicator.

Table 3a (LPM results) and Table 3b (Logit results) show the estimated effect of an increase of 1°C of the different means of temperatures on the prevalence of infected fish. All mean temperature parameters were significantly associated with infection prevalence (*p* < 0.001). For example, the predictions showed that a one-degree increment elevation in the mean temperature of June induces an increase of 6.9% of the prevalence of infected fish for the LPM [Table 3a, Model (1)] and an increase of 9.9% for the Logit [Table 3b, Model (1)].

**Table 3a:** Linear predicted increase/decrease of *T. bryosalmonae* infected fish prevalence due to an increase of 1 unit in the mean temperature variables and additional environmental parameters.

	**Model 1**	**Model 2**	**Model 3**	**Model 4**	**Model 5**
Mean temperature June	0.069^***^ (0.005)				
Mean temperature July		0.048^***^ (0.004)			
Mean temperature August			0.050^***^ (0.004)		
Mean temperature Summer				0.057^***^ (0.004)	
Total mean temperature					0.063^***^ (0.005)
IBCH (note)	0.006 (0.004)	0.013^**^ (0.005)	0.014^**^ (0.005)	0.012^**^ (0.004)	0.013^**^ (0.005)
WWTP	0.181^***^ (0.027)	0.230^***^ (0.026)	0.235^***^ (0.026)	0.216^***^ (0.026)	0.212^***^ (0.027)
Ecomorphology	−0.030^*^ (0.014)	0.001 (0.015)	0.002 (0.015)	−0.006 (0.015)	−0.014 (0.015)
N	1,068	1,068	1,068	1,068	1,068
R2	0.211	0.181	0.188	0.196	0.169

*Robust standard error in parentheses*.

* *p <0.10*,

** *p <0.05*,

*** *p <0.01*.

**Table 3b:** Logistic predicted increase/decrease of *T. bryosalmonae* infected fish prevalence due to an increase of 1 unit in the mean temperature variables and additional environmental parameters.

	**Model 1**	**Model 2**	**Model 3**	**Model 4**	**Model 5**
Mean temperature June	0.099^***^ (0.007)				
Mean temperature July		0.065^***^ (0.006)			
Mean temperature August			0.069^***^ (0.006)		
Mean temperature Summer				0.084^***^ (0.007)	
Total mean temperature					0.100^***^ (0.010)
IBCH (note)	0.006 (0.004)	0.016^***^ (0.004)	0.019^***^ (0.004)	0.016^***^ (0.004)	0.020^***^ (0.004)
WWTP	0.190^***^ (0.025)	0.225^***^ (0.024)	0.235^***^ (0.024)	0.213^***^ (0.084)	0.206^***^ (0.250)
Ecomorphology	−0.021^*^ (0.012)	0.021^*^ (0.012)	0.026^*^ (0.012)	0.015 (0.012)	0.001 (0.012)
*N*	1,068	1,068	1,068	1,068	1,068
Pseudo R2	0.373	0.299	0.315	0.339	0.296

*Robust standard error in parentheses*.

* p < 0.10, ^**^ p < 0.05,

*** *p < 0.01*.

The temperature parameter that explained at best the variance of the prevalence of infected fish (R2) was obtained with the model using the mean temperature of June [Table 3a, Model (1), R2 = 0.211] followed by the model taking into account the mean temperature of summer [Model (4), R2 = 0.196] for the LPM. The same findings appeared for the Logit [Table 3b, Model (1), Pseudo R2 = 0.373; Model (4), Pseudo R2 = 0.339]. Table 4a (LPM) and Table 4b (Logit) give the quantification of the average influence of the N days ≥ x°C and other environmental factors on the prevalence of the disease. Every temperature variable was significant (*p* < 0.01). For the LPM model, the N days ≥ 16 appeared to be the best explanatory temperature variable (R2 = 0.208), followed by the N days ≥ 15 (R2 = 0.203). The N days ≥ 14 (Pseudo R2 = 0.313) and then the N days ≥ 15 (Pseudo R2 = 0.309) had the higher Pseudo R2 for the Logit models. Therefore, all models showed that an increase in water temperature induced an increment in the predicted prevalence of infected fish. For the mean temperature category, the mean temperature of June and the mean temperature of the summer period explained at best the variance of the infection prevalence, both LPM and Logit combined. For the number of days with a daily mean temperature ≥ x°C, the LPM and Logit did not result in the same findings for the best explanatory variable for the variance of PKD prevalence (respectively, N days ≥ 16 and N days ≥ 14) but were coincident for the second explanatory variable (N days ≥ 15). Variance of each parameter was better explained with the Logit model than with the LPM model. However, R2 and Pseudo R2 remained low for every model.

**Table 4a:** Linear predicted increase/decrease of *T. bryosalmonae* infected fish prevalence due to an increase of 1 unit in the in the number of days with a daily mean ≥ x°C variables and additional environmental parameters.

	**Model 1**	**Model 2**	**Model 3**	**Model 4**	**Model 5**	**Model 6**	**Model 7**	**Model 8**
Days with daily mean ≥ 13°C	0.004^***^ (0.000)							
Days with daily mean ≥ 14°C		0.004^***^ (0.000)						
Days with daily mean ≥ 15°C			0.005^***^ (0.000)					
Days with daily mean ≥ 16°C				0.007^***^ (0.001)				
Days with daily mean ≥ 17°C					0.012^***^ (0.001)			
Days with daily mean ≥ 18°C						0.016^***^ (0.003)		
Days with daily mean ≥ 19°C							0.035^***^ (0.007)	
Degree days								0.004^***^ (0.000)
IBCH (note)	0.012^*^ (0.005)	0.012^**^ (0.004)	0.014^**^ (0.004)	0.012^**^ (0.004)	0.004 (0.004)	−0.001 (0.004)	−0.004 (0.004)	0.015^**^ (0.005)
WWTP	0.222^***^ (0.026)	0.200^***^ (0.026)	0.191^***^ (0.027)	0.221^***^ (0.026)	0.265^***^ (0.026)	0.272^***^ (0.026)	0.290^***^ (0.026)	0.209^***^ (0.026)
Ecomorphology	−0.002 (0.015)	−0.002 (0.015)	−0.009 (0.015)	−0.024 (0.015)	−0.028 (0.015)	−0.02 (0.015)	−0.016 (0.016)	−0.018 (0.015)
*N*	1,068	1,068	1,068	1,068	1,068	1,068	1,068	1,068
R2	0.18	0.194	0.203	0.208	0.191	0.148	0.133	0.171

*Robust standard error in parentheses*.

* *p <0.10*,

** *p <0.05*,

*** *p <0.01*.

**Table 4b:** Logistic predicted increase/decrease of *T. bryosalmonae* infected fish prevalence due to an increase of 1 unit in the in the number of days with a daily mean ≥ x°C variables and additional environmental parameters.

	**Model 1**	**Model 2**	**Model 3**	**Model 4**	**Model 5**	**Model 6**	**Model 7**	**Model 8**
Days with daily mean ≥ 13°C	0.005^***^ (0.000)							
Days with daily mean ≥ 14°C		0.005^***^ (0.000)						
Days with daily mean ≥ 15°C			0.005^***^ (0.000)					
Days with daily mean ≥ 16°C				0.006^***^ (0.001)				
Days with daily mean ≥ 17°C					0.010^***^ (0.001)			
Days with daily mean ≥ 18°C						0.013^***^ (0.002)		
Days with daily mean ≥ 19°C							0.031^***^ (0.005)	
Degree days								0.005^***^ (0.001)
IBCH (note)	0.015^**^ (0.004)	0.017^***^ (0.004)	0.017^***^ (0.004)	0.014^***^ (0.004)	0.005 (0.004)	0.001 (0.004)	−0.002 (0.004)	0.021^***^ (0.004)
WWTP	0.225^***^ (0.024)	0.203^***^ (0.024)	0.201^***^ (0.025)	0.234^***^ (0.025)	0.282^***^ (0.026)	0.285^***^ (0.026)	0.308^***^ (0.027)	0.204^***^ (0.028)
Ecomorphology	0.017 (0.012)	0.022^*^ (0.012)	0.015 (0.012)	−0.003 (0.012)	−0.013 (0.013)	−0.019 (0.014)	−0.015 (0.014)	−0.001 (0.001)
*N*	1,068	1,068	1,068	1,068	1,068	1,068	1,068	1,068
Pseudo R2	0.299	0.313	0.309	0.304	0.277	0.214	0.201	0.298

*Robust standard error in parentheses*.

* *p <0.10*,

** *p <0.05*,

*** *p <0.01*.

Concerning the additional environmental parameters, the IBCH index was significantly associated with PKD prevalence (*p* < 0.05) only in Logit models (four out of five models dealing with means of temperature and in five of the eight models of the number of days with a daily mean ≥ x°C). An increase of one unit in the IBCH score induced a predicted increase smaller than 2.1% in the PKD prevalence. The presence of an upstream WWTP was significantly linked to the disease (*p* < 0.01) in all models. The presence of such a facility increased the predicted PKD prevalence between 18 and 30.8% at a given site. On the contrary, ecomorphology was never significantly linked to PKD prevalence (*p* < 0.05). Therefore, presence of an upstream WWTP seemed to play a role in the predicted PKD prevalence, while IBCH score and ecomorphology had less influence.

**Figure 5:**
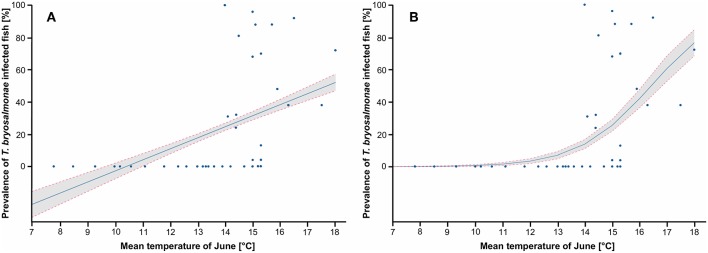
Prediction of the prevalence of *T. bryosalmonae* infected fish depending on the mean temperature of June. **(A)** Prediction of the linear probability model (LPM), R2 is 0.211. **(B)** Prediction of the logistic model (Logit), Pseudo R2 is 0.373. Blue points correspond to study sites, blue line corresponds to the prediction, gray zone corresponds to the 95% confidence intervals.

**Figure 6:**
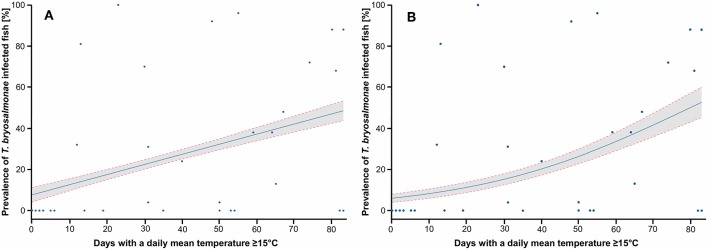
Prediction of the prevalence of *T. bryosalmonae* infected fish depending on the number of days with a daily mean temperature ≥ 15°C. **(A)** Prediction of the linear probability model (LPM), R2 is 0.203. **(B)** Prediction of the logistic model (Logit), Pseudo R2 is 0.309. Blue points correspond to study sites, blue line corresponds to the prediction, gray zone corresponds to the 95% confidence intervals.

Since both LPM and Logit models predicted that the mean temperature of June and the N days ≥ 15 explained well the variance of the infection prevalence, these two parameters were chosen for the following graphs. The LPM and Logit predicted PKD prevalence depending on the mean temperature of June, with study sites projection, are shown (Figures 5A,B). Infected fish were detected in stations with a mean temperature in June of 14°C at least. LPM predicted negative prevalence for temperature below 10°C, which highlighted limits of the model. On the contrary, the prediction issued from the Logit better corresponds to the observations. The predicted prevalence started to be positive from a mean temperature of June at 13°C and then increased. This model predicted a marked threshold effect from which infected fish may be found in the wild. The predictions of LPM and Logit models in function of the N days ≥ 15 are shown (Figures 6A,B). Infected fish were observed at sites with a mean temperature of ≥15°C for at least 12 days. The difference between the LPM and Logit model was weaker compared to the prediction of the mean temperature of June.

## Discussion

We performed a large-scale field investigation with longitudinal temperature measurements to demonstrate that temperature does indeed influence PKD prevalence and intensity in the wild. The possible combined influence of additional environmental parameters upon disease dynamics was also tested. Moreover, we developed a statistical model to highlight the predicted increase of PKD prevalence with rising water temperatures and/or additional environmental factors. Our analysis confirmed a positive relationship existing between water temperature and PKD prevalence and intensity in wild trout. The mean temperature of June and the N days ≥ 15 were the two parameters that were most strongly associated with PKD prevalence. The mean temperature of the summer period (June to August) was also a strong indicator for the prevalence of infected fish. Depending on the models, the macroinvertebrate index might sometimes have a positive correlation with the disease prevalence. The presence of an upstream WWTP showed significant aggravating influence on the infection prevalence, while the ecomorphology had less importance. Logistic model had a better explanatory power than linear model. However, the variance of the models remained low, suggesting that other parameters than the factors tested here might also have an influence on the disease. Thus, our findings (1) increased the knowledge of the implication of water temperature in PKD prevalence and intensity by investigating the impact of long-term temperature data, with seasonal, diurnal and site-specific changes, on PKD infection, in a large number of field sites within a specific region, (2) determined other disease modulating factors, such as water quality, and (3) quantitatively predicted the further impact of environmental parameters on the given prevalence.

### PKD Manifestation

In our study, PKD infected fish were generally detected in the downstream part of the rivers, while stations close to the source were free of the disease, as observed in the Boiron (upstream sites BOI011 and BOI013 were PKD free, while infected fish were found from the site RTV028 and downstream), the Venoge (no infected fish were observed in the site RTV055 but *T. bryosalmonae* positive fish appeared from station RTV054 and downstream) and the Veyron (upstream site RTV061 was free of the disease while parasites were found in downstream stations RTV060 and RTV085). Based on a study of 287 sampling sites throughout Switzerland, Wahli et al. (38) observed that the disease was present at an altitude lower than 800 m in most cases. Feist et al. (65) observed PKD prevalence ranging from 0 to 43% in England, which corresponds also to the findings of Peeler et al. (66) (PKD prevalence ranging from 2.5 to 36%). In our study, PKD prevalence ranged from 0 to 100% at a scale level of one canton, as observed by Wahli et al. (38) at a scale of a country. Therefore, PKD prevalence is also highly spatially variable, even within the same region.

### Relationship Between Temperature and PKD Infection

The influence of water temperature on PKD infection has been investigated mostly in laboratory with rainbow trout as model species, but studies comprising *in situ* water temperature data sets over seasons and their impact on wild brown trout populations are scarce. Wahli et al. (38) correlated PKD prevalence with altitude (as a substitution for temperature) in a wide variety of sites spread all over Switzerland, but they found no relationship between these two elements. On the contrary, in this study, a relationship between longitudinal temperature data and PKD prevalence and intensity was assessed within a field setting. *T. bryosalmonae* infection might appear when temperature reaches 9°C (36). Clinical signs and mortality are enhanced when the water temperature is >15°C (19, 23, 25, 39). Bettge et al. (18) showed that renal pathology of rainbow trout and parasite numbers were more intense at elevated temperature. Immune response strategy chosen by rainbow trout is also dependant of water temperature (37). Moreover, high temperature results in an accelerated rate for parasite release of brown trout, modifying therefore the parasite transmission period (33).

Our results do not support the findings of Gay et al. (36) since infected fish were observed in sites with warmer temperature than 9°C, even if trout were also sampled in colder streams. However, Gay et al. used rainbow trout as model species and experiments were performed in the laboratory. We also observed infected fish in sites with a minimum of 12 days with a daily mean of ≥15°C, which is slightly < the 14 to 28 days at 15°C predicted by Fischnetz (67) for PKD development in the wild. Lewisch et al. (68) found a significant increased number of *T. bryosalmonae* infected brown trout in Austria at sampling sites comprising a minimum of 115 days with at least 1 hourly maximum water temperature measurement exceeding 15°C. However, no long-term water temperature measurements at the precise sampling sites were used for these two studies, which might explain the differences with our results.

Based on statistical analysis, we observed that, when comparing the LPM and Logit, the Logit seemed to better fit to the data. This model should therefore be favored for further investigations. We found that the explanatory power of the variance from the mean temperature of summer (Pseudo R2 = 0.373) was also very close to the mean temperature of June results (Pseudo R2 = 0.339). Therefore, for a more global approach, the mean temperature of the summer period might also be a good indicator, instead of a precise month. Indeed, monthly temporal variations are more likely to depend in a particular year than the mean of the whole summer season. Taking into account the summer mean temperature is therefore a possibility to reduce the influence of a specific month, in particular for year to year comparisons. Given the water temperature regime of a stream is known, our results permit to assess if the thermal conditions are reached for the development of the infection or to estimate the predicted increase of PKD prevalence following rising water temperature due to global warming. Solutions to prevent this increase of water temperature might therefore be proposed to counteract the possible spread of the disease. Our model estimated that an increase of one degree in the mean temperature of summer might result in an increase of 5.7% in infection prevalence. Therefore, with rising temperature in Switzerland (69–71) due to the ongoing global warming (72), the PKD infection prevalence might intensify in already infected zones possibly leading to the parasite colonizing new areas, thus, extending the geographic range of *T. bryosalmonae* (16, 19, 29). This phenomenon is also relevant for other emerging diseases sensitive to temperature (28, 30), such as furunculosis caused by the bacterium *Aeromonas salmonicida* (73) or enteric redmouth disease due to *Yersinia ruckeri* (74).

### The Relationship Between Additional Environmental Factors and PKD Infection Prevalence

Studies have shown that low water quality can induce a decrease in freshwater biodiversity, such as macroinvertebrate community (56, 75, 76) or fish diversity (77, 78). In this study, we investigated the combined effect of different environmental factors and their relationship with PKD disease dynamics. Our analyses revealed that PKD prevalence was not strongly influenced by the state of the macroinvertebrate community measured by means of the Swiss biological index “IBCH,” which assessed water quality depending on the presence or absence of taxon sensitive to pollutants, as stoneflies for example. The presence of macroinvertebrate community is also driven by other factors, such as hydrology and habitat of a stream and does not focus only on water pollutants. The benthic fauna might therefore not be an ideal predictor for the disease presence.

We also analyzed the presence of an upstream WWTP as in indicator for water quality. Schmidt et al. (79) explored the effect of a sewage plant effluent on rainbow and brown trout health. When comparing trout kept in tap water and fish raised in water with an input of water coming from the WWTP, they found some alterations in internal organs but nothing that highlighted a real impact of low water quality on the decrease of brown trout populations. Bernet et al. (80) observed a decrease in brown trout health due to wastewater effluent. El-Matbouli and Hoffman (47) investigated the influence of water quality on *T. bryosalmonae*-rainbow and brown trout system. A significant difference in the PKD prevalence from sampled trout upstream and downstream of a WWTP effluent was observed. Significant differences in PKD prevalence and parasite intensity also appeared between two environmentally similar sites distant of 400 m, upstream and downstream of a WWTP in Switzerland (31). Our results corroborate these findings, since the presence of a WWTP appeared to be a significant aggravating parameter. Indeed, PKD prevalence increased by 20% if there was a WWTP present. However, the present study as well as the abovementioned referenced reports do not allow to assess if the decrease in water quality influences the disease prevalence either by directly affecting the fish health making them more vulnerable to the parasite infection, or influenced indirectly *T. bryosalmonae* infection by favoring the presence of bryozoans, since polluted water seems to favor the proliferation of filter feeding bryozoans (19, 36) which, in turn, increases the number of parasites in water. For instance, Hartikainen et al. (81) showed that an increase in nutrient concentration promotes bryozoan biomass and growth rates of *Fredericella sultana*, the most common bryozoan hosting *T. bryosalmonae*. Moreover, the concentration of statoblast was significantly promoted by phosphorus concentrations. A possibility for discriminating these two hypotheses would be to test the direct impact of water quality on fish health by performing a controlled experiment with infected trout raised in good and low water quality. Therefore, even if water quality has been considerably improved by sewage treatment in Switzerland (60) and the majority of WWTP effluent respect the Swiss legislation (82), we found that WWTP effluents play a role in the infection and might thus be considered when studying PKD in the wild. However, the variable used here was “presence” or “absence” of a WWTP, therefore more detailed investigations of this factor should be conducted, such as taking into account the dilution ratio of the effluent in the stream, the distance between the WWTP and the study site or the inhabitant equivalent of WWTP, which might reveal further information on the influence of WWTP effluents.

We observed that ecomorphology was not a discriminant factor for PKD prevalence. Thus, this parameter does not seem to act directly on the infection. However, this factor might characterize the habitat complexity of the stream, which could impact the trout density (61–63). Moreover, a stream with canalized riverbanks without trees could lead to higher water temperature. With rising temperature due to global change, this situation could become even worse. Therefore, especially in the downstream part of anthropized rivers, some habitats are susceptible to turn thermally unsuitable for brown trout, which could lead to a population decrease (6), as suspected in the southern periphery of brown trout distribution range (83–85). Thus, restoration measures should be taken in rivers, as planting trees along open streams, which will thus reduce the temperature increase by making shadow or remove physical barriers to restore the migration to upstream thermal refuges.

## Conclusion

Our results highlight the combined influence of water temperature, water quality and ecomorphology on PKD prevalence and intensity in wild brown trout populations. Water temperature appeared to be the major factor for PKD prevalence. When the necessary water temperature conditions are reached, the presence of an upstream WWTP might also play a role. These findings are crucial, especially in the context of global warming. Indeed, an increase in summer mean temperature and temperature variability might have consequences in the *T. bryosalmonae*-brown trout system, together with the potential geographical expansion of PKD. Therefore, knowing the significant parameters influencing the infection will allow to identify and to predict the potential disease hot spots in the future. Solutions to prevent this temperature increase and actions against the further spread of the disease might be proposed in terms of river management, mainly in downstream parts of river where water temperatures are higher and water quality is reduced.

## Data Availability

The datasets generated for this study are available on request to the corresponding author.

## Ethics Statement

This study was carried out in accordance with the recommendations of the Swiss Confederation. The protocol of macrozoobenthos sampling was approved by the Service of Hunting, Fishing, and Surveillance of the Biodiversity and Landscape Division from the General Directorate of the canton of Vaud, and the protocol of fish sampling was approved by the Service of Consumption and Veterinary business of the canton of Vaud (permission number VD2871).

## Author Contributions

AR, J-FR, and TW conceived the experiment. PdC, AR, and J-FR performed the sampling. PdC and AR carried out the laboratory analysis. CB, PdC, AR, J-FR, HS, and TW analyzed the data. AR wrote the draft of the paper. All authors commented on the manuscript.

### Conflict of Interest Statement

The authors declare that the research was conducted in the absence of any commercial or financial relationships that could be construed as a potential conflict of interest.

## Abbreviations

PKD: Proliferative kidney disease
IBCH: Swiss biological index
WWTP: Wastewater treatment plant
N days ≥ x: Number of days with a daily mean temperature ≥ x°C.
